# Construction and Immunological Evaluation of a Bivalent DNA Vaccine Targeting *Micropterus salmoides* Rhabdovirus and Largemouth Bass Ranavirus

**DOI:** 10.3390/ani16162551

**Published:** 2026-08-15

**Authors:** Boge Gu, Pengzhen Yu, Jinhao Su, Changhong Li, Jianfei Lu, Jiong Chen

**Affiliations:** 1 Laboratory of Biochemistry and Molecular Biology, School of Marine Sciences, Ningbo University, Ningbo 315211, Chinalichanghong@nbu.edu.cn (C.L.); 2 Key Laboratory of Aquacultural Biotechnology of Ministry of Education, Ningbo University, Ningbo 315211, China

**Keywords:** *Micropterus salmoides*, *Micropterus salmoides* rhabdovirus, largemouth bass ranavirus, DNA vaccine, bivalent vaccine

## Abstract

The largemouth bass (*Micropterus salmoides*) is persistently threatened by *Micropterus salmoides* rhabdovirus (MSRV) and largemouth bass ranavirus (LMBV), highlighting the need for combined immunization strategies. Here, a bivalent DNA vaccine pEGFP-G-MCP was constructed by concatenating the highly immunogenic fragments of MSRV glycoprotein (MSRV-G) and LMBV main capsid protein (LMBV-MCP) using a flexible linker peptide, and its immunogenicity and protective effect were systematically evaluated. Fish were immunized by intramuscular injection of 200 ng of plasmid on day 1, followed by a booster immunization on day 8. Blood and tissues were collected at 10, 20, and 30 d post-last immunization. The results showed that pEGFP-G-MCP immunization induced G- and MCP-specific antibodies and upregulated the expression of immunity-related genes. The bivalent vaccine achieved a relative percent survival (RPS) of 93.3% against MSRV and 90.0% against LMBV, which were higher than those of the monovalent vaccine groups. Additionally, the bivalent vaccine reduced viral mRNA and alleviated pathological damage in MSRV- or LMBV-infected fish. These results indicate that pEGFP-G-MCP confers immune protection against MSRV and LMBV, providing a foundation for combined prevention and control of MSRV and LMBV.

## Simple Summary

Largemouth bass are constantly threatened by MSRV and LMBV, while monovalent vaccines show limited prevention efficacy. Compared with monovalent vaccines, bivalent vaccines can simplify the immunization process, reduce fish pressure, and lower costs. In this study, a bivalent DNA vaccine pEGFP-G-MCP was constructed by fusing immunogenic fragments of MSRV-G and LMBV-MCP. Immunization induced specific antibodies and upregulated immune genes. The vaccine achieved relative percent survivals of 93.3% against MSRV and 90.0% against LMBV, exceeding monovalent groups. These results confirm that pEGFP-G-MCP confers robust protection, offering a foundation for integrated prevention of both pathogens.

## 1. Introduction

*Micropterus salmoides* rhabdovirus (MSRV) and largemouth bass ranavirus (LMBV) are two major viral pathogens that threaten largemouth bass (*Micropterus salmoides*) aquaculture [1]. MSRV, a member of the family *Rhabdoviridae*, is a single-stranded, negative-sense RNA virus that primarily infects juvenile fish, causing abdominal distension and fin base hemorrhage, with mortality exceeding 90% in acute outbreaks [2,3]. LMBV belongs to the genus *Ranavirus* within the family *Iridoviridae* and is a large double-stranded DNA virus, and mortality rates can reach 86.7% [4]. Notably, natural co-infection with both viruses has been documented in diseased fish, resulting in cumulative mortality as high as 93.3%, significantly exceeding that of single-pathogen infections [4]. Infected fish exhibit cutaneous ulceration and muscle necrosis [1].

Vaccination is an effective strategy for controlling viral diseases in aquaculture [5]. Among available vaccine platforms, DNA vaccines offer several key advantages, including flexible design, rapid construction, ease of production and transport, and a favorable safety profile. At present, DNA vaccines have demonstrated promising efficacy in multiple fish virus models [6,7,8]. Under intensive farming conditions, co-infection of pathogens can weaken the protective efficacy of monovalent vaccines [9]. Multivalent vaccines expand the protection spectrum while simultaneously reducing the cost and labor associated with preparation and administration [10,11]. Therefore, the development of combined immunization strategies capable of controlling multiple key pathogens within a single vaccination regimen is of considerable practical value.

The MSRV glycoprotein (MSRV-G) mediates receptor recognition and membrane fusion and is the principal target of neutralizing antibodies [12,13]. Truncated MSRV-G retains immunodominant epitopes, making it easier to expose antigen recognition sites, thereby conferring enhanced immunogenicity relative to the full-length protein [14]. In previous studies, the 382–795 bp region of MSRV-G was identified as a high antigenicity fragment that induced stronger protective immunity than full-length MSRV-G [14]. The main capsid protein (MCP) of LMBV is the core structural component of viral particles, with a relatively conserved sequence and good immunogenicity [15]. The 343–714 bp region of MCP was identified as an immunodominant region and induced stronger protection than the full-length MCP [16].

This is the first fusion DNA vaccine designed to protect largemouth bass against both MSRV and LMBV. Here, we constructed the bivalent DNA plasmid pEGFP-G-MCP by fusing the high-antigenicity regions of MSRV-G and LMBV-MCP via a flexible linker peptide and systematically evaluated its immunogenicity and protective efficacy in largemouth bass.

## 2. Materials and Methods

### 2.1. Bacterial Strains, Cells, and Pathogens

The MSRV strain SY01 (GenBank No.: PV339936.1) was isolated in May 2023 from Shangyu, Zhejiang Province, China [17], and the LMBV strain NB001 (GenBank No.: MN176304) was isolated in July 2018 from Ningbo, Zhejiang Province, China [18]. *Micropterus salmoides* dorsal fin (MSDF) cells were cultured in M199 medium (Gibco, Grand Island, NY, USA) supplemented with 15% fetal bovine serum (FBS) at 25 °C. HEK293T cells were cultured in DMEM (Gibco) supplemented with 10% FBS at 37 °C with 5% CO_2_. The pEGFP-N1 vector was purchased from Clontech (Mountain View, CA, USA). *Escherichia coli* DH5α was maintained in our laboratory.

Viruses were propagated in MSDF cells. When the cytopathic effect reached approximately 90%, cells and supernatant were harvested, subjected to three freeze–thaw cycles, and centrifuged (7000× *g*, 20 min) [19]. The supernatants were filtered through a 0.22 μm filter and stored at −80 °C. Virus titration was performed using the standard 50% tissue culture infective dose (TCID_50_) assay. The supernatant containing MSRV or LMBV was collected for further study.

### 2.2. Construction of Recombinant Plasmid

Total RNA was extracted from MSDF cells infected with MSRV using TRIzol reagent (TaKaRa, Osaka, Japan) and reverse transcribed into cDNA by PrimeScript™ II 1st Strand cDNA Synthesis Kit (Takara). Briefly, total RNA (1 μg) was denatured with oligo(dT) and dNTPs at 65 °C, then chilled on ice. First-strand cDNA was synthesized using RTase at 42 °C for 60 min, followed by inactivation at 70 °C for 15 min.

The genomic DNA was extracted from MSDF cells infected with LMBV using a genomic DNA extraction kit (Tiangen, Beijing, China). The 382-795 bp fragment of MSRV-G (GenBank No.: XQO28573.1) was analyzed using IEDB B cell linear epitope prediction (Bepipred 2.0, default threshold 0.5) and DNASTAR 11 (antigenic index, hydrophilicity, flexibility, and surface probability; performed in May 2025). The 382-714 bp fragment was found to be a highly antigenic fragment among the 382-795 bp fragments. The truncated MCP fragment (343-714 bp; GenBank No.: MN176304) was from a previous study [16]. Primers were listed in Table 1. The amplified G and MCP products were verified by agarose gel electrophoresis and Sanger sequencing.

For monovalent plasmids, double enzyme cleavage was performed using *HindIII* and *BamHI* (TaKaRa), followed by ligation with T4 DNA ligase (TaKaRa) to obtain recombinant plasmids pEGFP-G and pEGFP-MCP.

For bivalent plasmids, overlap extension PCR was used to concatenate G and MCP fragments, connected by a flexible peptide linker (GGGGSGGGGS; nucleotide sequence: GGCGGTGGCGGTAGCGGCGGTGGCGGTAGC). The G-MCP fusion fragment was cloned into the pEGFP-N1 vector using the pEASY^®^-Basic Seamless Cloning and Assembly Kit (TransGen, Beijing, China), yielding the recombinant plasmid pEGFP-G-MCP. The reverse orientation plasmid pEGFP-MCP-G was constructed using the same strategy. All recombinant plasmids were confirmed by Sanger sequencing. These recombinant plasmids can fuse the target protein with GFP for expression.

### 2.3. Expression of Recombinant Plasmids

The pEGFP-N1, pEGFP-G, pEGFP-MCP, pEGFP-G-MCP, and pEGFP-MCP-G were individually transfected into HEK293T cells. Briefly, HEK293T cells were seeded in 6-well plates and cultured to 80–90% confluence. Plasmid DNA (2.5 μg/well) was transfected using Lipofectamine 8000 (5 μL/well; Beyotime, Hangzhou, China). Transfections were performed in triplicate (three independent experiments). At 24 h post-transfection, the fluorescence was observed under a fluorescence microscope (Nikon, Tokyo, Japan). The excitation/emission wavelengths were 488 nm/509 nm, respectively. All images were captured under identical exposure conditions. Fluorescence intensity was quantified using ImageJ 1.54f from three random fields per sample.

### 2.4. Western Blotting (WB)

Cell lysates were fractionated by 12% SDS-PAGE and transferred onto a 0.45 μm PVDF membrane (Merck Millipore, Darmstadt, Germany). The membrane was blocked with 5% bovine serum albumin (BSA) for 2 h at room temperature and incubated overnight at 4 °C with GST Mouse Monoclonal Antibody (1:1000; Beyotime). After washing with PBST, the membrane was probed with HRP-conjugated goat anti-mouse IgG (1:3000; Beyotime) for 2 h. Protein bands were detected using an ECL Western Blotting Substrate (Thermo Fisher, Waltham, MA, USA).

### 2.5. Immunization and Sample Collection

Juvenile largemouth bass were obtained from a commercial farm in Suzhou, Jiangsu Province, and acclimated for two weeks at the aquaculture facility of Ningbo University. Fish were maintained in 200 L tanks with water temperature at 23 ± 1 °C, dissolved oxygen ≥ 6.0 mg/L, pH 7.4 ± 0.2, and ammonia nitrogen ≤ 0.02 mg/L. Water was partially exchanged every 2–3 days (~1/5–2/5 of the whole volume) with feces removed by siphon. After acclimation, fish with a body weight of 2 ± 0.2 g and a body length of 4.0 ± 0.3 cm were selected. During the experimental period, fish were fed commercial compound feed twice daily. Liver and spleen tissues were collected and tested by PCR to confirm the absence of MSRV and LMBV infection.

For the immunization procedure, fish were randomly assigned to five groups (3 replicates per group, with 100 fish raised in a 200 L tank for each replicate) using a random number generator-based grouping method: PBS group, pEGFP-N1 group, pEGFP-G monovalent vaccine group, pEGFP-MCP monovalent vaccine group, and pEGFP-G-MCP bivalent vaccine group. The plasmid was diluted in PBS to a concentration of 20 ng/μL and administered via intramuscular injection at the dorsal fin base at a dose of 200 ng/fish (10 μL per fish), as previously described [28]. Fish were immunized on day 1 and received a booster immunization on day 8. The tank served as the biological replicate unit.

Collect blood, liver, and spleen tissues on days 10, 20, and 30 after the booster immunization. Randomly select 9 fish from each group and mix them into a sample every 3 fish, with 3 replicates in each group. Sampled fish were excluded from subsequent analyses. Blood was collected from the tail vein using a syringe, allowed to stand overnight at 4 °C, and then centrifuged at 2000× *g* for 15 min to obtain serum. Serum samples were stored at −80 °C until use.

### 2.6. ELISA

G- and MCP-specific antibodies in serum were measured by indirect ELISA. Serum samples obtained in Section 2.5 were diluted 1:200 and coated onto 96-well plates (100 μL/well, *n* = 3) in duplicate, followed by overnight incubation at 4 °C. Plates were washed three times with PBST and blocked with 5% BSA for 2 h at 37 °C. Purified G or MCP (1 μg/well, 100 μL), expressed and purified as previously described [23,29], was added and incubated for 1 h. After washing, the anti-G protein polyclonal antibody (1:500), previously generated and validated in our laboratory [23], or the mouse anti-LMBV MCP monoclonal antibody (1:500; Biogoethe, Wuhan, China) was added and incubated for 1 h at 37 °C. Plates were then incubated with HRP-conjugated goat anti-mouse IgG antibody (1:5000, 100 μL/well) for 1 h at 37 °C. TMB chromogenic substrate (Tiangen) was added and allowed to develop for 15 min. The reaction was stopped with ELISA stop solution (Solarbio, Beijing, China), and absorbance was measured at 450 nm, and absorbance was measured by a microplate reader. All assays were performed in duplicate, and *n* = 3 represents 3 biological replicates per group.

### 2.7. Immune-Related Gene Expression

The expression of immune-related genes was measured by real-time quantitative PCR (RT-qPCR) as previously described [30]. Target genes included the humoral immunity marker *immunoglobulin M* (*IgM*); innate antiviral genes *cyclic GMP-AMP synthase* (*cGAS*), *interferon regulatory factor 3* (*IRF3*), *interferon regulatory factor 7* (*IRF7*), *interferon 1* (*IFN1*), *myxovirus resistance 2* (*Mx2*); inflammation-related genes *interleukin-1β* (*IL*-*1β*), *tumor necrosis factor α* (*TNF*-*α*), and *interleukin-27* (*IL*-*27*).

Total RNA was extracted using TRIzol reagent. Reverse transcription was performed using HiScript III All-in-one RT SuperMix Perfect for qPCR (Vazyme, Nanjing, China) with 200 ng of total RNA (50 °C for 15 min, 85 °C for 5 s). qPCR was performed in a 10 μL reaction volume containing 200 ng cDNA, qPCR Mix, and primers on a Real-Time PCR System (ABI, Foster City, CA, USA). Thermal cycling conditions were: 95 °C for 30 s, followed by 40 cycles of 95 °C for 10 s and 60 °C for 30 s. Primer sequences were listed in Table 1. All samples underwent 3 technical replicates, and NTC/NRT controls were consistently negative. *18S rRNA* served as the internal reference gene, which has been validated as a stable reference in largemouth bass tissues [23,31,32], and relative expression levels were calculated using the 2^−ΔΔCt^ method.

### 2.8. Challenge Experiment and Survival Rate Analysis

For MSRV challenge, 50 fish were randomly selected from each group (excluding the pEGFP-MCP monovalent group) and intraperitoneally injected with 50 μL of MSRV (1 × 10^4.6^ TCID_50_/mL; ~2000 TCID_50_/fish). For LMBV challenge, fish were randomly selected from each group (excluding the pEGFP-G monovalent group) and intraperitoneally injected with 50 μL of LMBV (1 × 10^6.5^ TCID_50_/mL; ~1.6 × 10^5^ TCID_50_/fish). A healthy group (no challenge, but injected with the same dose of PBS) was maintained in parallel under identical rearing conditions to serve as a baseline reference for survival. For survival analysis, 30 fish from each tank (three tanks per group) were monitored for 15 d post-infection. Only fish that died with typical clinical signs of infection were recorded as dead. Survival data were plotted as Kaplan–Meier curves. For viral load quantification, liver and spleen tissues were collected at 48 h post-infection. In each experimental tank, three fish were randomly selected, and their tissues were pooled to generate one biological replicate; three such tanks were used per group. Similarly, three fish from each tank were randomly chosen for histopathological examination. All fish were euthanized by immersion in MS-222 at a dose of 0.1 g/kg body weight.

### 2.9. Viral Load Quantification

Total RNA was extracted and reverse-transcribed into cDNA, and the relative transcript levels of *MSRV-N* and *LMBV-MCP* were quantified by RT-qPCR as described in Section 2.7. *18S rRNA* was used as the internal reference.

### 2.10. Histopathology

Liver and spleen tissues were fixed in 4% paraformaldehyde (Biosharp, Hefei, China) for 48 h. Fixed tissues were dehydrated through a graded ethanol series, cleared in xylene, and embedded in paraffin. Sections were cut to a thickness of 5 μm, stained with hematoxylin and eosin (H&E), and examined under a light microscope (Leica, Wetzlar, Germany) to assess histopathological alterations. Liver and spleen tissues from healthy, uninfected fish were processed as controls.

Histopathological changes in the liver and spleen were graded on a 0–3 scale (0 = absent, 1 = mild, 2 = moderate, 3 = severe) as previously described [33].

### 2.11. Statistical Analysis

Data were analyzed and graphed using GraphPad Prism 10.1. Data were presented as mean ± standard deviation (SD). Normality was assessed by the Shapiro–Wilk test, and homogeneity of variances was assessed by Levene’s test. Multi-group comparisons were analyzed by one-way analysis of variance (ANOVA) followed by Tukey’s post hoc test. Survival curves were analyzed using the Mantel–Cox log-rank test. Hazard ratios (HRs) and 95% confidence intervals were calculated by Cox proportional hazards regression. Differences were considered statistically significant at * *p* < 0.05, ** *p* < 0.01, and *** *p* < 0.001.

## 3. Results

### 3.1. Expression of Recombinant Proteins

**Figure 1:**
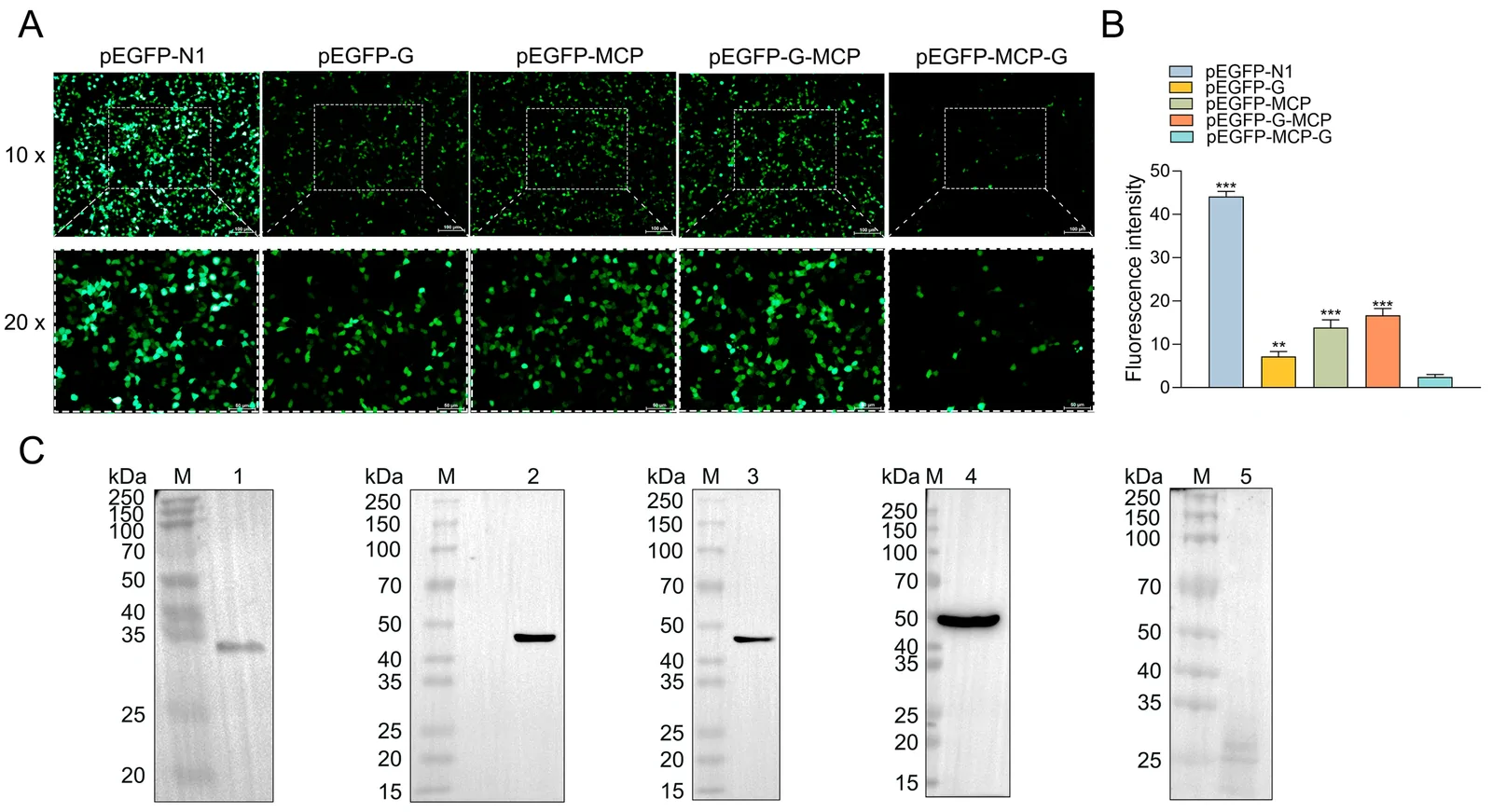
Expression of recombinant proteins in HEK293T cells. pEGFP-N1, pEGFP-G, pEGFP-MCP, pEGFP-G-MCP, and pEGFP-MCP-G plasmids were transfected to HEK293T cells. (**A**) EGFP fluorescence (green) was observed at 24 h post-transfection. The upper row shows the photo taken under a 10× microscope, and the lower row shows the photo taken under a 20× microscope. (**B**) Mean GFP fluorescence intensity was quantified by ImageJ from three random fields per sample. (**C**) WB results of recombinant proteins. Line 1, pEGFP-N1; Line 2, pEGFP-G; Line 3, pEGFP-MCP; Line 4, pEGFP-G-MCP; Line 5, pEGFP-MCP-G. Data were presented as mean ± SD. Statistical analysis was performed by one-way ANOVA followed by Tukey’s post hoc test. (*n* = 3). ** *p* < 0.01, *** *p* < 0.001. All significance analyses were conducted using the pEGFP-MCP-G group as the reference.

Green fluorescence was detected in all the transfected HEK293T cells at 24 h post-transfection. The fluorescence in pEGFP-MCP-G transfected cells was weak, while the fluorescence in pEGFP-G-MCP transfected cells was strong (Figure 1A). Mean GFP intensity (a.u.) quantified confirmed that pEGFP-G, pEGFP-MCP, and pEGFP-G-MCP exhibited higher expression than pEGFP-MCP-G (Figure 1B). WB results found specific bands corresponding to the expected molecular weights were detected in the pEGFP-N1 (~27 kDa), pEGFP-G (~39 kDa), pEGFP-MCP (~41 kDa), and pEGFP-G-MCP (~54 kDa), whereas no obvious band was observed in the pEGFP-MCP-G group (Figure 1C). Therefore, pEGFP-G-MCP was chosen for further research.

### 3.2. Vaccination Induces Antigen-Specific Serum Antibodies

**Figure 2:**
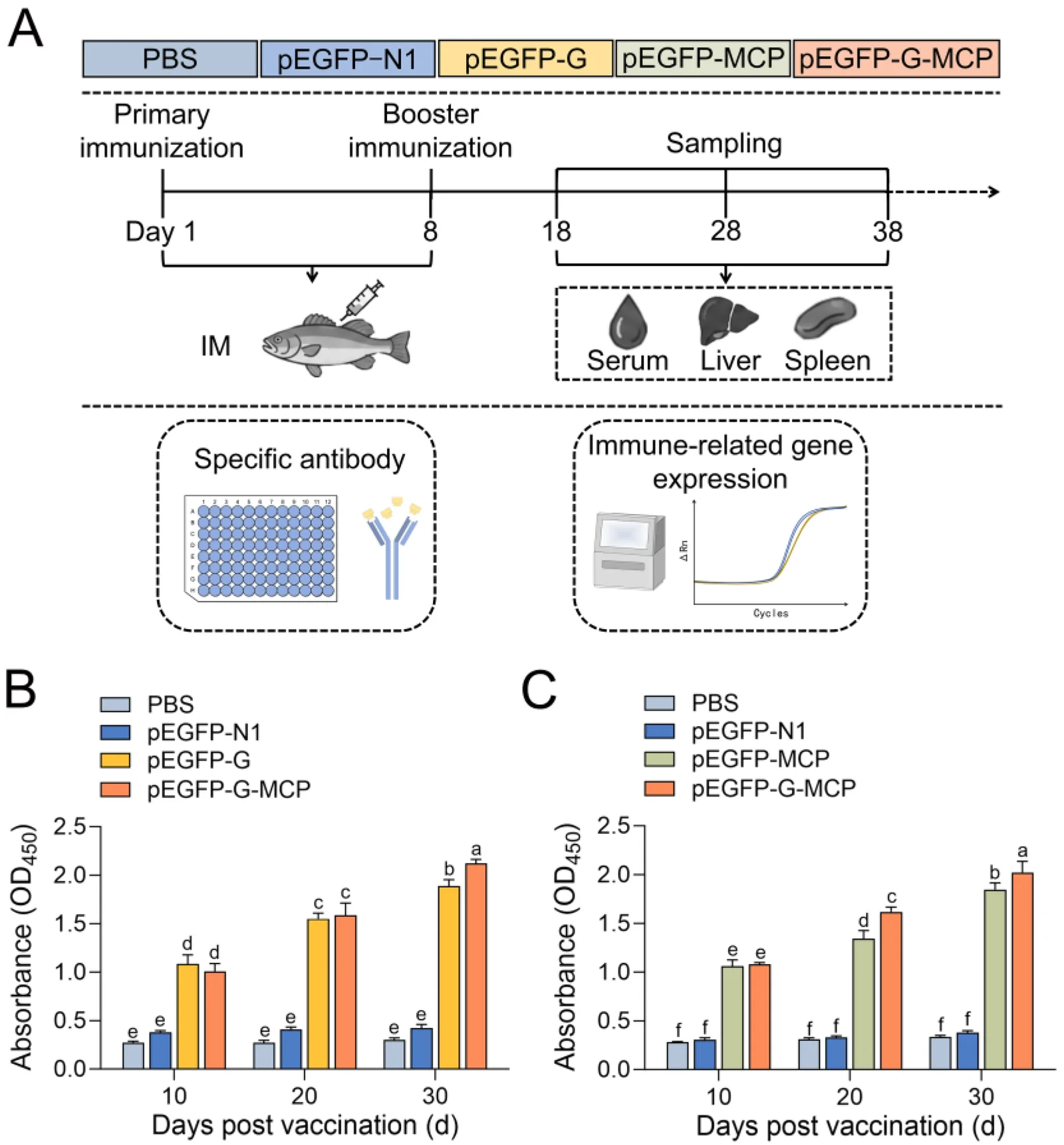
Immunization protocol and specific antibody detection in largemouth bass. (**A**) Schematic diagram of animal grouping and immunization protocol. (**B**) G-specific antibody detected by ELISA. (**C**) MCP-specific antibody detected by ELISA. Data were presented as mean ± SD (*n* = 3). Statistical analysis was performed by one-way ANOVA followed by Tukey’s post hoc test. Different letters above bars indicate significant differences (*p* < 0.05); the same letter indicates no significant difference. All significance analyses were conducted using the pEGFP-N1 control group as the reference.

The prepared DNA vaccine was immunized according to the procedure shown in Figure 2A. Both the pEGFP-G monovalent group and the pEGFP-G-MCP bivalent vaccine induced G-specific antibody, which increased over time (Figure 2B). The highest G-specific antibody levels appeared at 30 d in the pEGFP-G-MCP group. Similarly, both the pEGFP-MCP monovalent and the bivalent vaccine induced MCP-specific antibody in a time-dependent manner (Figure 2C). The highest MCP-specific antibody levels also appeared at the 30th day of the pEGFP-G-MCP group.

Direct comparisons between groups showed that G-specific antibody levels in the bivalent group were higher than those in the pEGFP-G monovalent group at 30 d, and MCP-specific antibody levels were higher than those in the pEGFP-MCP monovalent group at 20 d. No significant differences were observed at other time points.

### 3.3. Vaccine Induces Immune-Related Gene Expression in the Liver

**Figure 3:**
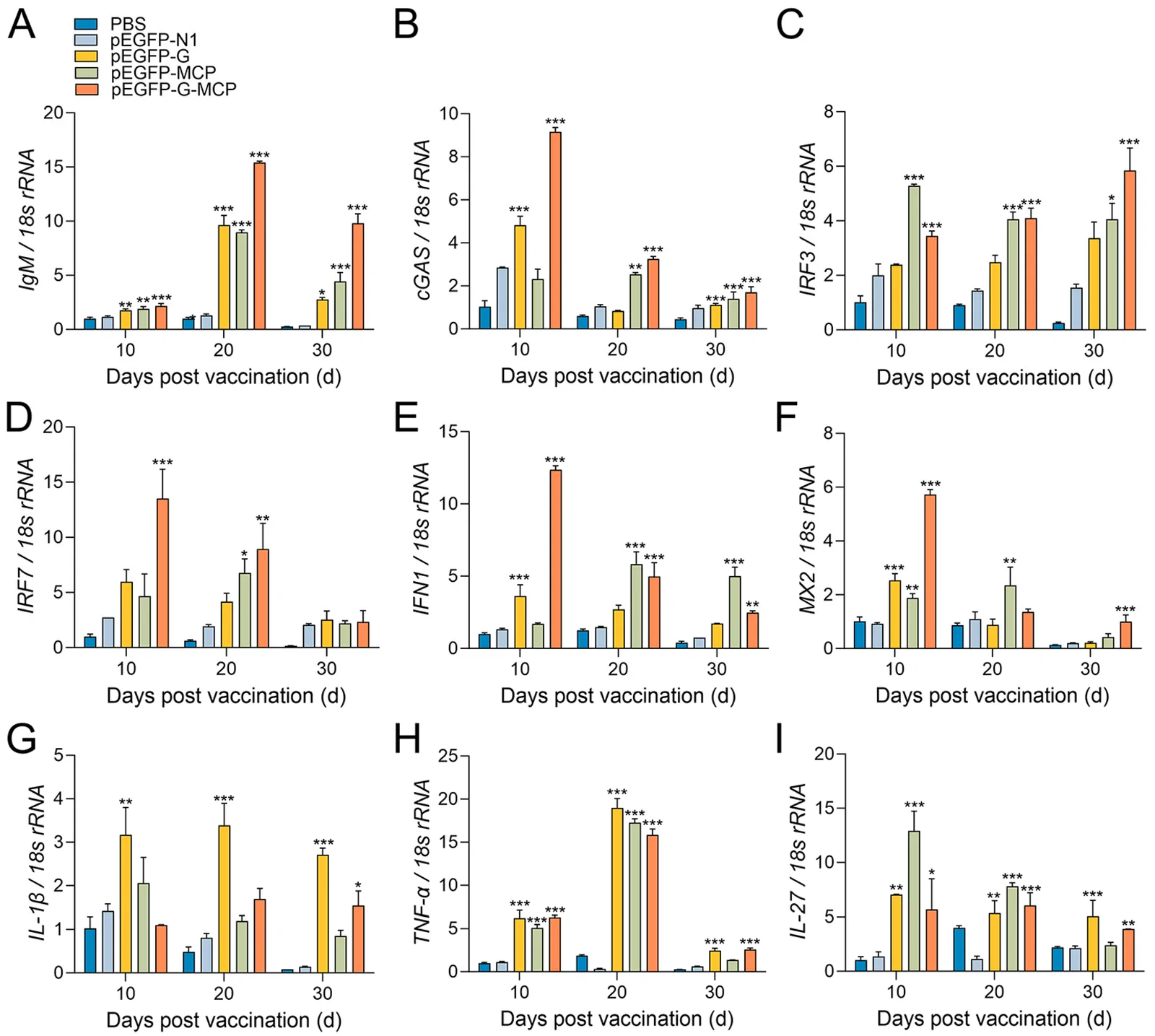
Immune-related gene expression in the liver of largemouth bass. mRNA levels of *IgM* (**A**), *Cgas* (**B**), *IRF3* (**C**), *IRF7* (**D**), *IFN1* (**E**), *Mx2* (**F**), *IL*-*1β* (**G**), *TNF*-*α* (**H**), and *IL*-*27* (**I**) were determined by RT-qPCR. Data were presented as mean ± SD (*n* = 3). Statistical analysis was performed by one-way ANOVA followed by Tukey’s post hoc test. * *p* < 0.05, ** *p* < 0.01, *** *p* < 0.001. All significance analyses were conducted using the pEGFP-N1 control group as the reference.

At 10, 20, and 30 d after the last immunization, the mRNA levels of 9 immune-related genes in the liver were determined by RT-qPCR (Figure 3). In the liver, *IgM* transcription was upregulated in all vaccine groups compared with the pEGFP-N1 group. The highest *IgM* level appeared in the pEGFP-G-MCP bivalent group 20 d after the last immunization, at 15.4-fold (Figure 3A). *cGAS*, *IRF3*, *IRF7*, *IFN1*, and *Mx2* genes were upregulated across all vaccine groups (Figure 3B–F). *IL*-*1β* and *TNF*-*α* levels were transcriptionally upregulated across all vaccine groups relative to controls. Notably, *IL*-*1β* and *TNF*-*α* exhibited the peak expression values in the pEGFP-G group (Figure 3G,H). *IL*-*27* was also upregulated across all vaccine groups in the liver, with the strongest induction observed in the pEGFP-MCP group (Figure 3I).

### 3.4. Vaccine Induces Immune-Related Gene Expression in the Spleen

**Figure 4:**
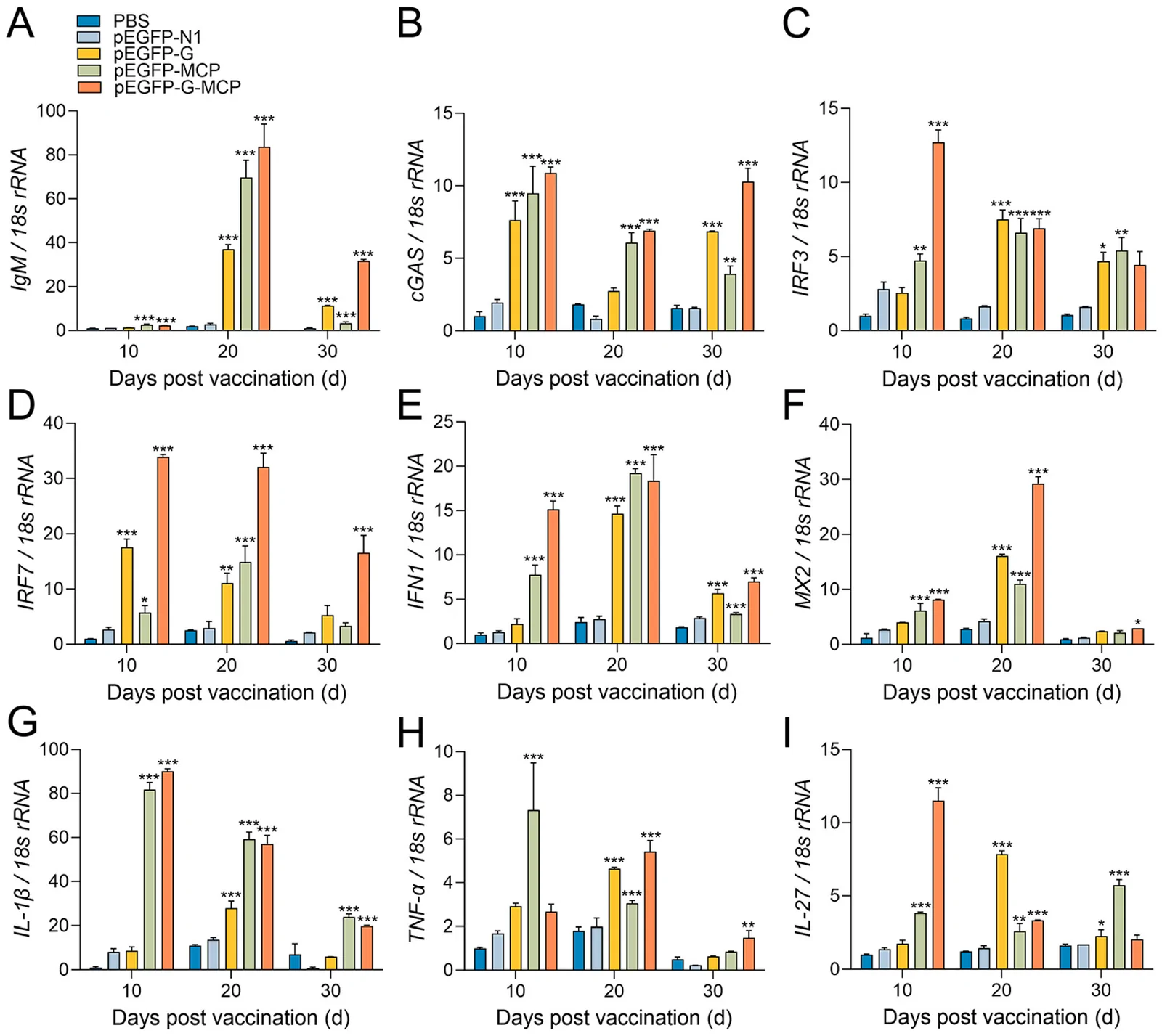
Immune-related gene expression in the spleen of largemouth bass. mRNA levels of *IgM* (**A**), *Cgas* (**B**), *IRF3* (**C**), *IRF7* (**D**), *IFN1* (**E**), *Mx2* (**F**), *IL*-*1β* (**G**), *TNF*-*α* (**H**), and *IL*-*27* (**I**) were determined by RT-qPCR. Data were presented as mean ± SD (*n* = 3). Statistical analysis was performed by one-way ANOVA followed by Tukey’s post hoc test. * *p* < 0.05, ** *p* < 0.01, *** *p* < 0.001. All significance analyses were conducted using the pEGFP-N1 control group as the reference.

In the spleen, all the immune-related genes were upregulated. *IFN1* induction was most pronounced in the pEGFP-MCP group rather than the bivalent group. The cytokine responses also differed: *IL*-*1β* peaked in the bivalent group, *TNF*-*α* in the pEGFP-MCP group, and *IL*-*27* in the pEGFP-G-MCP group (Figure 4).

### 3.5. The Combined Vaccine Protects Fish from MSRV and LMBV Infection

**Figure 5:**
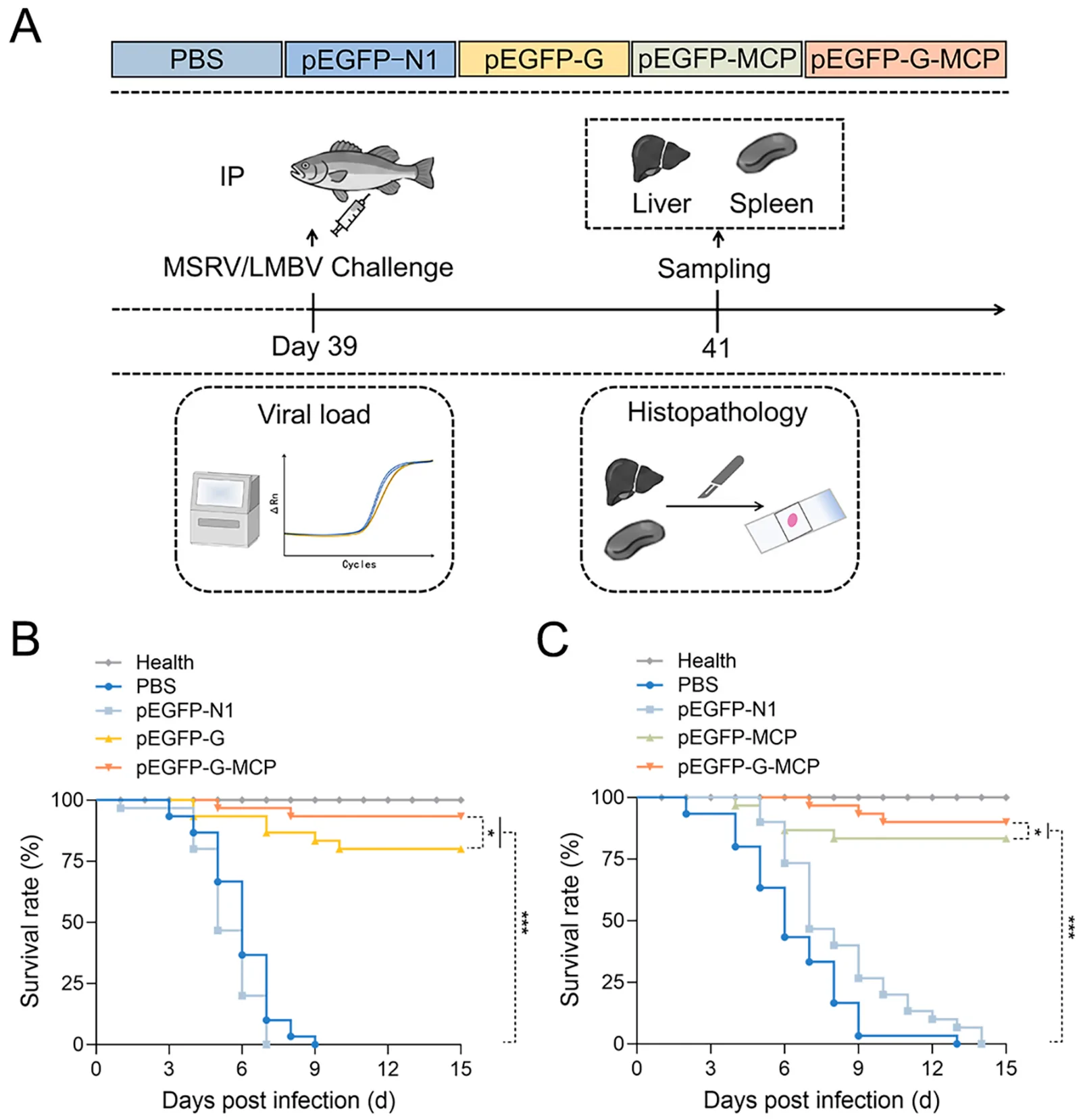
Schematic diagram of the challenge protocol and survival rate in largemouth bass. (**A**) Schematic diagram of the challenge protocol. (**B**) Survival curves of fish post-MSRV challenge. (**C**) Survival curves of fish post-LMBV challenge. Survival curves were analyzed using the log-rank test with pairwise comparisons. *n* = 3. * *p* < 0.05, *** *p* < 0.001. All significance analyses were conducted using the pEGFP-N1 control group as the reference.

In the MSRV challenge experiment, the mortality rates of the PBS and pEGFP-N1 control groups reached 100% on day 9 and day 7, respectively. Mortalities in the pEGFP-G group began on day 4, with a cumulative mortality of 20% (6/30) and a relative percent survival (RPS) of 80%. In the pEGFP-G-MCP group, mortalities began on day 5, with a cumulative mortality of 6.7% (2/30) and an RPS of 93.3%. Both the pEGFP-G and pEGFP-G-MCP groups exhibited higher RPS than the pEGFP-N1 group (*p* < 0.001), and the RPS of the pEGFP-G-MCP group was also higher than that of the pEGFP-G group (*p* < 0.05) (Figure 5B).

In the LMBV challenge experiment, the PBS and pEGFP-N1 control groups reached 100% mortality by day 13 and day 14, respectively. The pEGFP-MCP group showed mortalities from day 4, with a cumulative mortality of 16.7% (5/30) and an RPS of 83.3%. The pEGFP-G-MCP group showed mortalities from day 7, with a cumulative mortality of 10% (3/30) and an RPS of 90.0% (Figure 5C). The RPS of the pEGFP-MCP and pEGFP-G-MCP groups was higher than that of the pEGFP-N1 group (*p* < 0.001). Moreover, the pEGFP-G-MCP group exhibited a markedly higher RPS than the pEGFP-MCP group (*p* < 0.05) (Figure 5C).

### 3.6. Vaccine Reduces Viral Transcript Levels in Tissue Post-Infection

**Figure 6:**
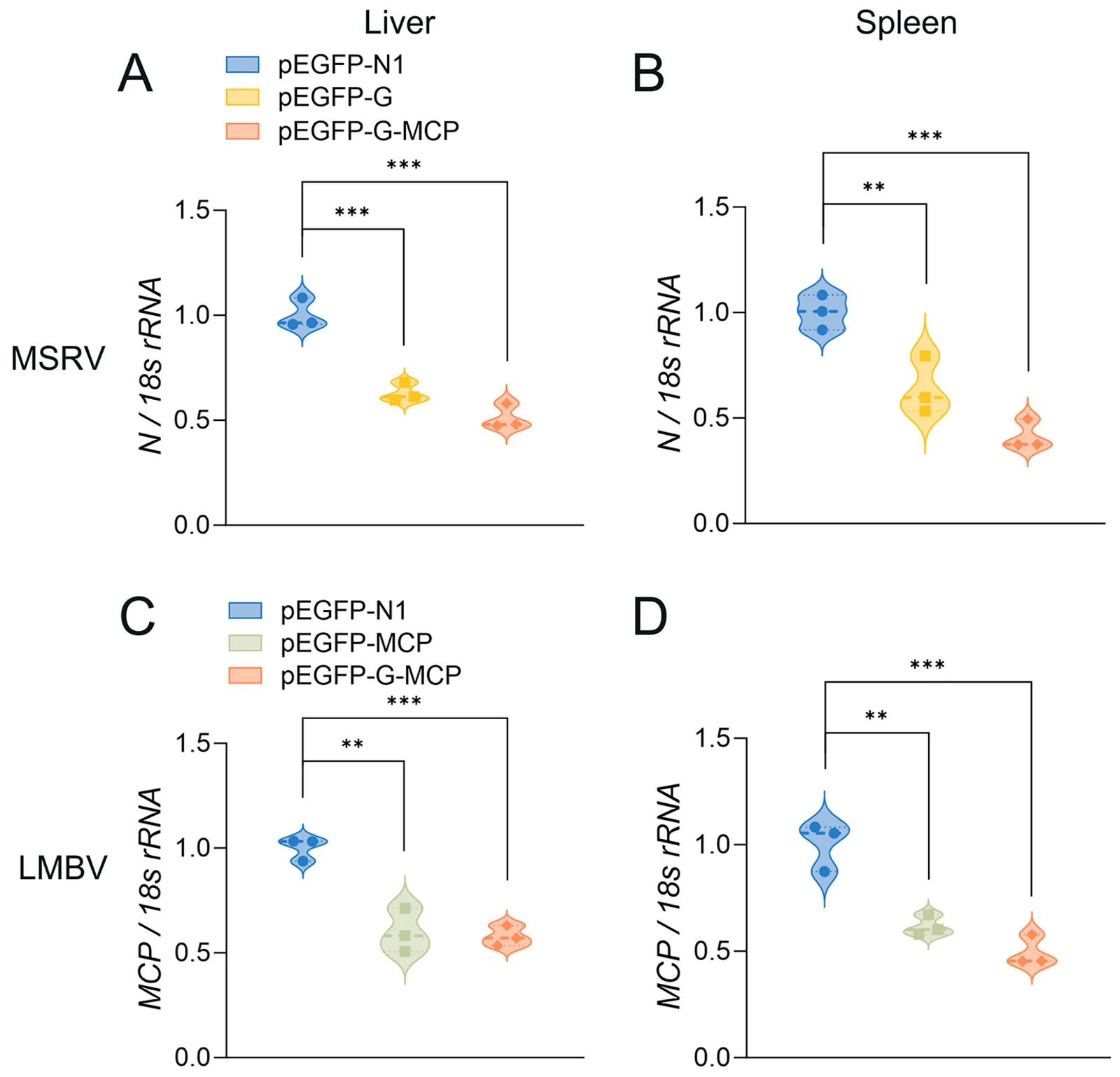
Viral transcript levels in the liver and spleen of largemouth bass. (**A**,**B**) Viral RNA levels following MSRV challenge. (**C**,**D**) Viral MCP transcript abundance following LMBV challenge. Data were presented as mean ± SD (*n* = 3). pEGFP-N1 group was used as a control for the 2^−ΔΔCt^ calculation. Statistical analysis was performed by one-way ANOVA followed by Tukey’s post hoc test. ** *p* < 0.01, *** *p* < 0.001.

The procedure of the challenge experiments was shown in Figure 5A. At 48 h post-challenge, viral transcript abundance in the tissues was quantified by RT-qPCR. Following the MSRV challenge, *MSRV-N* mRNA in both the pEGFP-G and pEGFP-G-MCP groups was lower than that in the pEGFP-N1 group (Figure 6A,B). In the liver, the bivalent group reduced *MSRV-N* mRNA by 48.87% relative to the pEGFP-N1 group, compared with a 37.11% reduction in the monovalent group. In the spleen, reductions of 58.6% and 36.0% were observed in the bivalent and monovalent groups, respectively.

Following the LMBV challenge, viral MCP transcript abundance in both the pEGFP-MCP and pEGFP-G-MCP groups was lower than that in the pEGFP-N1 group (Figure 6C,D). In the liver, the bivalent group reduced *LMBV-MCP* mRNA by 42.19% relative to the control, compared with a 40.02% reduction in the monovalent group. In the spleen, reductions of 50.7% and 38.4% were observed in the bivalent and monovalent groups, respectively.

### 3.7. Vaccine Attenuates Histopathological Damage

**Figure 7:**
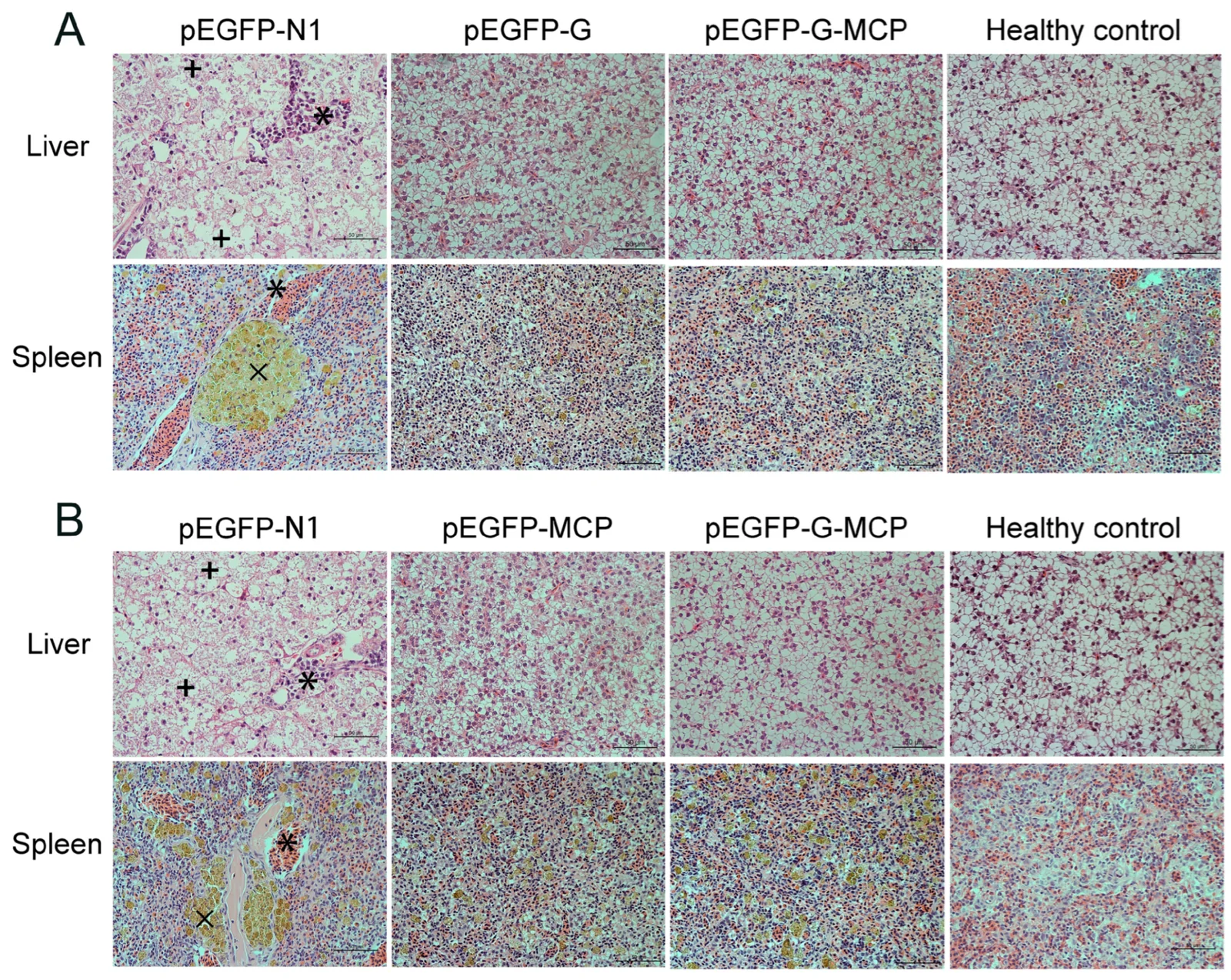
H&E staining of the liver and spleen. (**A**) Liver and spleen following MSRV challenge. (**B**) Liver and spleen following LMBV challenge. +: vacuolar degeneration; ×: hemosiderin deposition; *: hyperemia. Scale bar = 50 μm.

Following the MSRV challenge, the liver of the pEGFP-N1 control group exhibited diffuse vacuolar degeneration and hemosiderin deposition with hyperemia in the spleen (Figure 7A). Lesion scores were lower than those in the pEGFP-G-MCP group and the pEGFP-N1 group. Monovalent vaccine groups displayed a trend toward reduced histopathological damage relative to controls (Table 2).

Following the LMBV challenge, the pEGFP-N1 control group displayed similar pathological changes: diffuse vacuolar degeneration in the liver and hemosiderin deposition with hyperemia in the spleen (Figure 7B). Lesion scores were lower in the pEGFP-G-MCP group than in the pEGFP-N1 group in both the liver and spleen, whereas the pEGFP-MCP group did not reach statistical significance. Healthy control fish scored 0 in all tissues (Table 2). These results indicate that the bivalent DNA vaccine pEGFP-G-MCP attenuates virus-induced histopathological damage.

## 4. Discussion

Infections with MSRV and LMBV can cause mass mortality in largemouth bass, leading to substantial economic losses [1]. Despite the availability of monovalent vaccines targeting MSRV-G [12,14,23,34] and LMBV-MCP [24,29,35,36], no bivalent strategy has been developed to simultaneously protect largemouth bass against both viruses.

In the present study, the bivalent DNA vaccine we constructed (pEGFP-G-MCP) conferred significant protection against both MSRV and LMBV. The RPS against MSRV and LMBV were 93.3% and 90.0%, respectively, which were higher than those of the corresponding monovalent vaccines (80% and 83.3%) (Figure 5). This improved protective efficacy correlated well with the trends observed in neutralizing antibody titers (Figure 2), suggesting that the bivalent vaccine enhances antiviral protection by potentiating specific humoral immune responses. A plausible underlying mechanism is that the co-delivery of the two antigens triggers a more robust innate immune response than the single-antigen stimulation of monovalent vaccines. The combined antigens likely accelerate the recruitment and activation of immune cells within lymphoid tissues, providing augmented co-stimulatory signals that facilitate B-cell proliferation and differentiation. This, in turn, promotes the clonal expansion of specific B-cell populations, leading to higher levels of specific antibodies [37,38]. Notably, the tandem co-immunization of the two antigens did not induce antigenic competition or immunosuppression; instead, it exhibited a clear synergistic immune-enhancing effect. Collectively, these findings highlight the superiority of our bivalent vaccine design and offer mechanistic insights into its enhanced protective performance.

Significantly upregulated transcription of *cGAS*, *IRF3*, *IRF7*, *IFN1*, *Mx2*, *IL*-*1β*, *TNF*-*α* and *IL*-*27* was detected following vaccination. As an intracellular nucleic acid sensor, cGAS initiates downstream signaling cascades upon recognition of viral nucleic acids, which activates transcription factors IRF3 and IRF7 and subsequently triggers the expression of type I interferon (IFN1) and its downstream antiviral effector gene Mx2 [39,40,41,42]. The upregulation of these genes is consistent with the involvement of the cGAS–IRF3/7–IFN1 axis, although transcriptional data alone do not confirm pathway activation or establish cell-intrinsic antiviral defense. We note that cGAS recognizes double-stranded DNA, and the plasmid backbone itself may contribute to innate immune activation as a known adjuvant effect of DNA vaccines; the pEGFP-N1 control group serves to partially account for this. In addition, elevated expression of pro-inflammatory cytokines IL-1β and TNF-α is consistent with a pro-inflammatory response, but does not independently demonstrate antigen-presenting cell activation or immune cell recruitment [43,44,45,46]. Notably, the upregulation of immunoregulatory *IL*-*27* may serve a negative-feedback role to constrain excessive inflammation, though this mechanism was not experimentally tested in this study [47,48,49]. Taken together, these transcriptional changes are consistent with coordinated innate immune activation that may contribute to the protective efficacy of the vaccine.

Upregulation of *IgM* gene expression was observed post-vaccination. IgM serves as the predominant immunoglobulin mediating systemic humoral immunity during primary immune responses in fish [50,51]. Antigen stimulation by the vaccine promotes the proliferation and differentiation of B lymphocytes, leading to enhanced *IgM* transcription, thereby promoting the secretion of antigen-specific antibodies. These observations are consistent with the induction of antigen-specific humoral immune responses. As the principal mediator of systemic humoral immunity in teleosts, IgM may contribute to viral neutralization and opsonization, although these functions were not directly measured in this study. Combined with the upregulated innate immune genes described above, pEGFP-G-MCP appears to induce a coordinated innate immune response that provides cytokine signals to support B cell activation and differentiation.

## 5. Conclusions

In summary, this study demonstrates that a truncated-antigen fusion-based bivalent DNA vaccine, pEGFP-G-MCP, confers protective immunity against both MSRV and LMBV under laboratory conditions. The bivalent vaccine achieved an RPS of 93.3% against MSRV and 90.0% against LMBV, both higher than those of the respective monovalent vaccines. The bivalent vaccine also reduced viral transcript abundance and alleviated pathological damage. These findings establish a laboratory proof-of-concept for dual-target DNA vaccination in largemouth bass.

## Figures and Tables

**Table 1:** Primers used in this study.

Gene	Accession Number	Primer Sequence (5′-3′)	Amplicon Size (bp)	Purpose
pEGFP-G	XQO28573.1	F: CCCAAGCTTTGGGCTACAATCAGCGAT R: CGGGATCCCGCATTCCCACCTCTCCGCAC	352	Cloning
pEGFP-MCP [16]	MN176304.1	F: CCCAAGCTTGAGCACGCCGCACTCTCG R: CGGGATCCCGTTCCAGATCTGTGGCCAC	391	
pEGFP-G-linker	XQO28573.1	F: CAGATCTCGAGCTCAAGCTTTGGGCTACAATCAGC R: CCACCGCCGCTACCGCCACCGCCCATTCCCACCTC	376	Homologous recombination
pEGFP-MCP-linker	MN176304.1	F: GCGGTAGCGGCGGTGGCGGTAGCGAGCACGCCGCA R: GTGGCGACCGGTGGATCCCGTTCCAGATCTGTGGC	415	
IgM [20]	MN871984.1	F: CTCAATGACCCCCCCTAA R: CAAGCCAAGACACCAAAA	278	RT-qPCR
cGAS [21]	XM038737916.1	F: AGAGGGCTGGCTGGGACG R: CCAACTATTCTTAGCAGGGACC	121	
IRF3 [22]	XM038735465.1	F: CTTTAAGGCGTGGGCCGAG R: CTCCTGATGCTGACTCATC	179	
IRF7 [22]	XM038706685.1	F: CACTGAGAACATTTATGAAAGA R: CTCATTTCCTGAGGGGAAC	106	
IFN1 [23]	XM038709291.1	F: TCTTGCTGTGGTTCAGTCGG R: GCGATTAAGTGTGGGGTTG	110	
Mx2 [24]	XM038696208.1	F: TAAAATGGCTGGGGTCGGGG R: CATTGCACGGAACGACCACC	218	
IL-1β [23]	XM038733429.1	F: CATGGAAGGCGGTAAAG R: TTGGGGCAACTGAGAGA	150	
TNF-α [25]	XM038710731.1	F: CTTCGTCTACAGCCAGGCAT R: TTTGGCACACCGACCTCACC	161	
IL-27 [26]	XM038729305.1	F: CCAACCTGAAACTTCTCACCGACT R: CCTTGGTGCTCTCAAATGGACCC	288	
18S rRNA [23]	JQ896299.1	F: GGACACGGAAAGGATTGACAG R: GTTCGTTATCGGAATTAACCAGAC	111	
MSRV-N [27]	MK397811.2	F: GCCCACATCGCATCATTCAC R: GTGGCAGAGTAAGGGGACAC	182	
LMBV-MCP	MN176304.1	F: ACTTGCGTCACTCCTGTTGT R: AGGCTCAGGGCGTAAGAGTA	212	

**Table 2:** Histopathological lesion scores of the liver and spleen.

Challenge	Tissue	pEGFP-N1	pEGFP-G	pEGFP-MCP	pEGFP-G-MCP	Healthy Control
MSRV	Liver	2 (2–3)	0 (0–1)	–	0 (0–1) *	0 (0–0)
MSRV	Spleen	2 (2–2)	1 (1–1)	–	1 (0–1) *	0 (0–0)
LMBV	Liver	2 (2–3)	–	1 (0–1)	0 (0–1) *	0 (0–0)
LMBV	Spleen	2 (2–3)	–	1 (1–2)	1 (0–1) *	0 (0–0)

Note: Statistical analysis was performed by the Kruskal–Wallis test followed by Dunn’s post hoc test. * *p* < 0.05. The significance analyses were conducted using the pEGFP-N1 group as the reference. “–” indicates the group was not included in the corresponding challenge. Healthy control: uninfected, untreated fish.

## Data Availability

The original contributions presented in this study are included in the article. Further inquiries can be directed to the corresponding authors.

## Abbreviations

The following abbreviations are used in this manuscript:

DNA	deoxyriboNucleic acid
MSRV	*Micropterus salmoides* rhabdovirus
LMBV	largemouth bass ranavirus
G	glycoprotein
MCP	main capsid protein
RPS	relative percent survival
CPE	cytopathic effect
MSDF	*Micropterus salmoides* dorsal fin cell line
RNA	ribonucleic acid
ELISA	enzyme-linked immunosorbent assay
RT-qPCR	real-time quantitative PCR
IgM	immunoglobulin M
cGAS	cyclic GMP-AMP synthase
IRF3	interferon regulatory factor 3
IRF7	interferon regulatory factor 7
IFN1	interferon 1
Mx2	myxovirus resistance 2
IL-1β	interleukin-1β
TNF-α	tumor necrosis factor α
IL-27	interleukin-27
